# Characteristics of helicopter hoist operations with intubated patients: a retrospective analysis of a Norwegian physician staffed SAR helicopter service

**DOI:** 10.1186/s13049-024-01289-9

**Published:** 2024-11-21

**Authors:** Sven Christjar Skaiaa, André Roslin, Torfinn Heggland, Sigurd Heian, Trond Elden, Øivind Åreskjold, Hanne Rikstad Iversen, Dag Stian Jakobsen, Mads Sabel, Per Olav Berve

**Affiliations:** 1 Division of Prehospital Services, Air Ambulance Department, Oslo University Hospital, Oslo, Norway; 2 Department of Anaesthesiology, Intensive Care Medicine and Surgical Services, Førde Central Hospital, Førde, Norway; 3 Department of Emergency Medicine and Pre-Hospital Services, St. Olav University Hospital, Trondheim, Norway; 4 Air Ambulance Department, Nordland General Hospital, Bodø, Norway; 5 Clinic of Prehospital Services, Air Ambulance Department, Stavanger Health Trust, Stavanger, Norway; 6 Emergency Department, Finnmark Hospital, Hammerfest, Norway; 7 RNoAF 330 Squadron Rygge, 1580 Rygge, Norway

**Keywords:** Hoisting, Winching, HSAR, HEMS, Helicopter, Prehospital, Intubation, RSI, Rescue

## Abstract

**Background:**

Timely medical management and evacuation of critically ill or injured patients from austere environments or maritime vessels is often achieved by helicopter hoist operations. When indicated, intubation is performed onsite to restore and sustain patient physiology and to facilitate safe transport. We aimed to describe the characteristics of helicopter hoist operations (HHOs) with intubated patients in a physician staffed SAR helicopter service and to identify learning points for future missions.

**Methods:**

The Norwegian national SAR database and local medical journal systems on six SAR helicopter bases were searched for data on hoisted intubated patients from January 2011 to April 2024.

**Results:**

From a total of 18,710 missions, we registered 2,423 helicopter hoist operations with patients as human external cargo. In 54 hoist operations (2%) the patients were intubated prior to hoisting. We observed an increasing number of both HHOs in general and HHOs with intubated patients over time. The intubated HHO patients were in an overall critical state, with a median NACA score of 6 and a median GCS of 3 before intubation. Trauma was the main cause of intubation (n = 32). Twenty-five patients presented with cardiac arrest, 13 of whom were hoisted with an ongoing mechanical chest compression device. During the hoist operation, 34 patients were ventilated manually, and 20 patients were connected to an automatic ventilator. Monitoring of vital parameters during hoisting varied from none to fully monitored patients including invasive arterial blood pressure. Twenty-eight patients, seven of whom presented with initial cardiac arrest, survived to hospital discharge.

**Conclusions:**

HHOs with intubated patients are rare but increasingly occurring events in our service. Owing to the infrequency, complexity and risk factors involved, these operations should be governed by specific operating procedures and trained regularly to be performed safely. HHOs with intubated patients represents a favourable alternative in situations where terrestrial transport is associated with significant time delay or additional risk to the patient or the rescuers.

## Background

An essential part of Helicopter Emergency Medical Services (HEMS) and Helicopter Search- and Rescue (HSAR) is access to, management and evacuation of patients located in complex environments and on maritime vessels. These locations are typically time consuming to access by other means, austere in nature, or both. Depending on the medical urgency or impending environmental threats, some accident sites are only timely accessible by direct insertion of health- and rescue personnel by a helicopter hoist operation (HHO) or a helicopter longline operation.

When indicated, endotracheal intubation is performed onsite to sustain resuscitation and physiology, and to facilitate safe transport. Although hoist extrication of an intubated patient may be an efficient evacuation method, it is a highly complex operation with inherent risk factors to both the patient and rescuers [1, 2].

The prevalence of HHOs with intubated patients as human external cargo (iHEC) worldwide is reported to be low, ranging from 2 to 6% of HHOs with HEC [3–11]. With the exception of a few case reports, few studies have been published on the management of these patients [12–15].

The aim of this study was to describe the characteristics of HHOs with iHEC in a physician staffed Norwegian SAR helicopter service and to present learning points for future missions.

## Methods

### Study setting

**Fig. 1:**
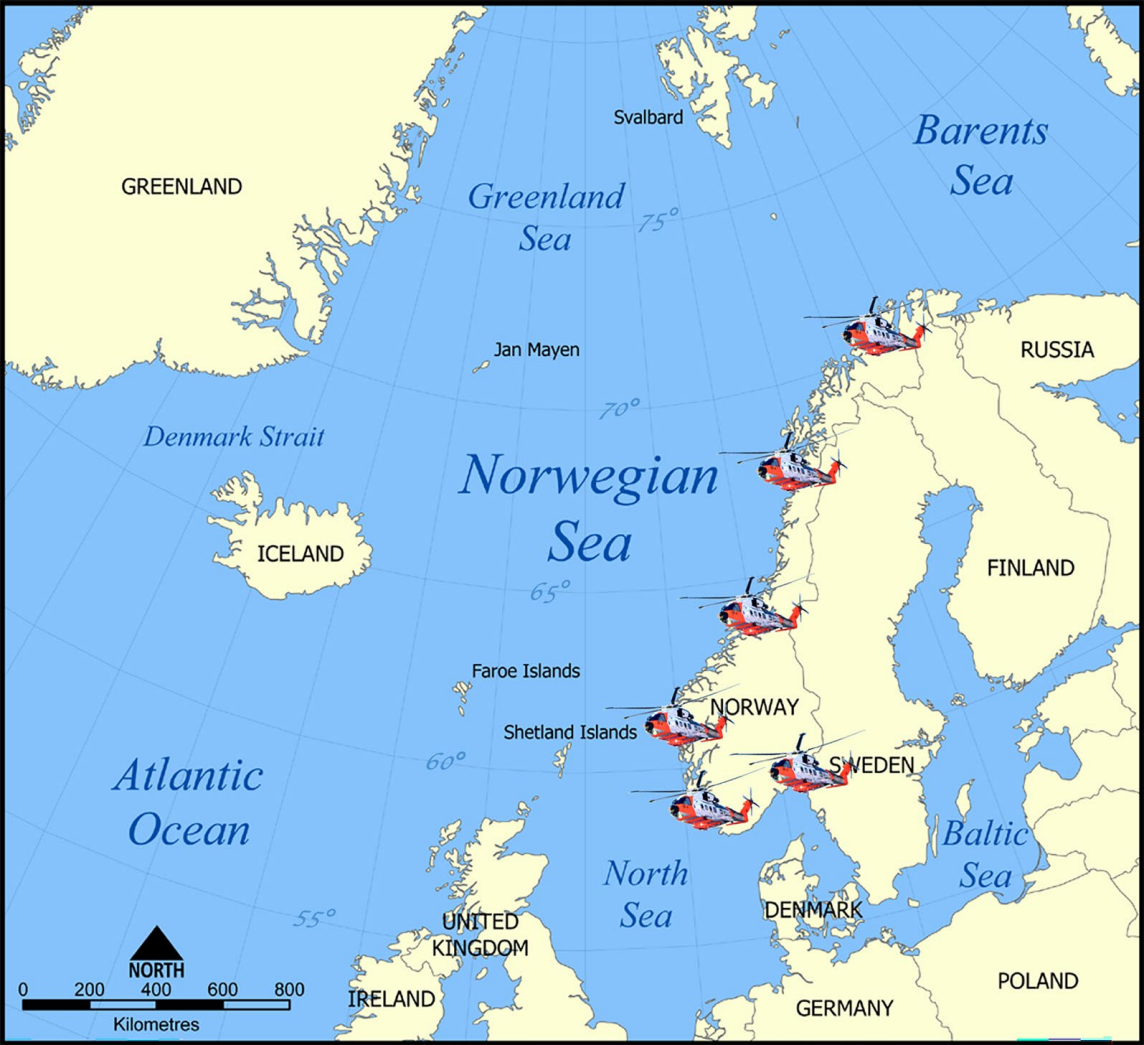
The RNoAF 330 Sqn SAR helicopter bases. One base was from 2017 to 2024 serviced by CHC Helikopter Service, Florø, Norway. Map source: www.wikipedia.com

The RNoAF 330 Sqn operates six SAR helicopter bases in Norway. Helicopter missions are dispatched by one of two Joint Rescue Coordination Centres (JRCC Southern Norway and JRCC Northern Norway). The bases are located along the Norwegian coastline from 57° N to 70° N, and 330 Sqn HSAR operations have spanned from 56° N in the southwestern part of the North Sea to 78° N in the Barents Sea (Fig. 1). Mission profiles vary between the bases due to differences in latitude, climate, topography, proximity to hospitals/EMS services, and population density (76,000 to 2.8 million) in the bases’ main operational area.

The 330 Sqn helicopter fleet consists of 15 Leonardo AW101 all-weather rescue helicopters. The helicopters are manned by six crew members consisting of two pilots, a hoist operator, a systems operator/navigator, a rescue specialist/swimmer (RS), and a consultant physician specializing in anaesthesiology, critical care medicine and HSAR operations. In addition, the RS retains medical qualifications as a registered nurse or paramedic. Each base employs four to five Air Force crewmembers of each category and six to eight civilian physicians.

While the RS is the primary rescue operator, the physician is trained to assist in technical rescue. The RS and physician hence work together as an interdisciplinary Rescue and Resuscitation Team (RRT). For potentially critical patients, and when landing is not feasible, the RRT is inserted close to the accident scene by hoist, including necessary medical equipment and a helicopter rescue bag or stretcher. For very complex or dangerous situations (including patients immersed in water) the RS might perform a direct hoist extrication without the physician. Advanced medical interventions will in this case be performed in the helicopter cabin.

For HHOs, the AW101 is equipped with two rescue hoists with an applicable hoist cable length of 88.4 m (290 feet) and a maximum working load of 272 kg (600 lbs).

HHOs do not always result in hoist extrication of a patient. Patients with minor injuries are sometimes handed over to terrestrial rescue teams or local EMSs. Others might, due to flight or patient safety, be transported over land or water to a parked helicopter or ambulance, and some are pronounced dead onsite.

Under acceptable flight conditions, and when ground transport is deemed either too time consuming or hazardous, patients are hoisted up into the helicopter as human external cargo (HEC). A hoist extrication of a patient takes approximately two minutes from the initiation of hoist ascent until the patient is established on the helicopter cabin stretcher. Patients with moderate to severe injury or illness are, if possible, accompanied by the physician during hoisting.

### Study design and data analysis

In this retrospective observational study, we present descriptive data on helicopter hoist operations with intubated patients. Background data and relevant missions were identified by conducting an electronic search in both the national SAR Database (SAR Rapport 2.0, Vikoyra industriomrade 7, 6230 Sykkylven, Norway) and each of the SAR helicopter bases’ patient journal systems (Labas 7, Normann IT, Norway). Because SAR database entries were not standardized before 2011, we included missions from January 1st, 2011, to April 30th, 2024. The search criteria were HSAR operations and the keywords “hoist” and “intubation or intubated”. Extracted HHO missions on each base were manually reviewed by the respective senior medical officer to identify cases that included iHEC. Mission data were thereafter transferred to Excel (Microsoft Excel version 16.87, MS365, Microsoft Corporation, USA) for analysis. The results are reported as numeric values with *n*, percentage and median with range.

## Results

### Mission profile

**Fig. 2:**
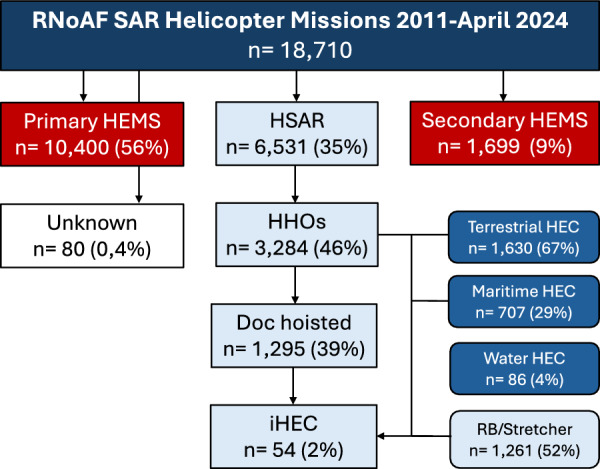
Study diagram for all helicopter missions 2011—April 2024. Of the HHOs, 2,423 mission resulted in HEC. The diagram illustrates from which environment the patients were hoisted: Terrestrial = Land/mountain/forest, Maritime = Boats and ships, Water = Direct extrication from ocean/lake/river. Of the terrestrial and maritime HEC, 1261 patients were hoisted horizontally in a helicopter rescue bag (RB) or stretcher

During the study period the HSAR bases completed 18,710 missions. A total of 10,400 (56%) were categorized as primary HEMS missions, 6531 (35%) as HSAR missions, and 1699 (9%) as secondary HEMS missions/interhospital transfers. Eighty missions (0.4%) lacked mission categorization (Fig. 2).

### Patient characteristics and medical interventions in all missions

The HSAR crew attended to 16,384 patients of whom 10,171 (62%) were male. Acute medical events were the main cause for dispatch in 8761 missions (54%), followed by 5474 traumatic causes (33%). The median NACA-score was 4 (range 0–7). CPR was performed on 1128 patients (7%), of whom a mechanical chest compression device (mCPR) was used in 430 patients (3%).

The most common intervention was oxygen (n = 6327, 39%), followed by crystalloid infusion (n = 4826, 30%) and analgesia (n = 3345, 20%). Advanced medical interventions included endotracheal intubation (n = 1818, 11%), total intravenous anaesthesia (n = 1248, 8%), automatic ventilation (n = 1106, 7%), invasive arterial blood pressure (IABP, n = 961, 6%) and blood product transfusion (n = 278, 2%).

An overview of patient characteristics and interventions is presented in Table 1.

**Table 1:** Patient characteristics and medical interventions for all helicopter missions 2011—April 2024 (n = 16,384)

Variable	n	%
Age, median decade age group	50–59	
< 18 years	1740	11
> 80 years	1314	8
Unknown/missing	1887	12
Gender		
Male	10,121	62
Female	5689	35
Unknown/missing	525	3
Diagnosis		
Medical	8761	53
Trauma	5474	33
Obstetric	588	4
Uninjured and unknown/missing	1581	10
NACA Score (National Advisory Committee for Aeronautics)		
0—No injury or illness	177	1
1—Injuries/diseases without any need for acute care by physician	334	2
2—Injuries/diseases requiring examination and therapy by physician, but hospital admission is not indicated	1045	6
3—Injuries/diseases without acute threat to life but requiring hospital admission	4424	27
4—Injuries/diseases that can possibly lead to deterioration of vital signs	4582	28
5—Injuries/diseases with acute threat to life	2178	13
6—Injuries/diseases transported after successful resuscitation of vital signs	1397	9
7—Lethal injuries or diseases (with or without resuscitation attempts)	1105	7
Unknown/missing	1142	7
Procedures and interventions		
Oxygen	6327	39
Crystalloid infusion	4826	30
Analgesia	3345	20
Other medication	2696	17
Endotracheal intubation	1818	11
Vasoactive drugs (IV/IO or infusion)	1807	11
Total intravenous anaesthesia (TIVA)	1248	8
Cardiopulmonary resuscitation (CPR)	1128	7
Automatic ventilator	1106	7
Invasive arterial blood pressure (IABP)	961	6
Fracture splinting	787	5
Point of care ultrasound (POCUS)	773	5
Mechanical chest compression device (mCPR)	430	3
Blood product transfusion	278	2
Intraosseous access	279	2
Central venous catheter	157	1
Tube thoracostomy	118	1

### Mission characteristics of HHOs with HEC

HHOs were performed in 3284 (50%) of the missions categorized as HSAR. While the RS was hoisted down in all HHOs, the physician was hoisted down in 1295 (39%). 2423 HHOs (74%) resulted in HEC. Of the HEC patients, 1261 (52%) were hoisted horizontally in a rescue bag or stretcher of which a total of 54 patients (2%) were identified as iHEC (Fig. 2). Among the iHEC missions, 27 (50%) occurred during the summer months (June–August), and 9 (17%) occurred during winter months (December-March). Fourteen missions (26%) were classified as night operations requiring night vision goggles. Thirty-seven patients (69%) were located on land or on a waterfront, and 17 (31%) were located on ships or boats. Twenty-two cases (41%) were categorized as sports related. An overview of mission characteristics is presented in Table 2.

**Table 2:** Mission characteristics of HHOs with iHEC 2011—April 2024 (n = 54)

Variable	n	%
Seasons and daylight		
Summer months (June–August)	27	50
Winter months (December–March)	9	17
Night missions (requiring night vision gear)	14	36
Location		
Terrain (mountain, forest)	26	48
Boat or ship	17	31
Waterfront	9	17
Urban area	2	4
Accident occurrence		
Hiking, running or cycling	8	15
Skiing	7	13
Boating and sports fishing	4	7
Driving	4	7
Occupational	4	7
Climbing	3	6
Air sports	2	4
Diving	2	4
Other (leisure, cruise ship tourism, self-harm)	20	37

**Fig. 3:**
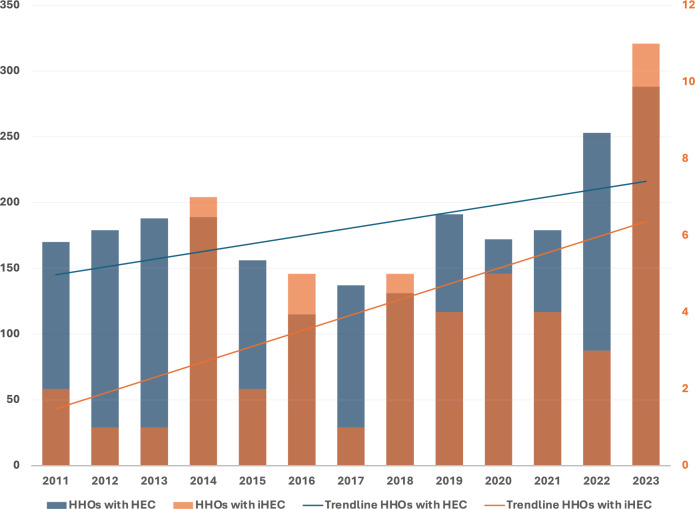
Incidence with trendlines for HHOs with HEC and iHEC in the RNoAF 330 Sqn 2011–2023

We observed an increasing frequency of iHEC missions during the study period. While 19 cases occurred during the first half of the study period, 35 cases occurred during the last half. In 2023, 11 cases of HHOs with iHEC were executed (Fig. 3).

### Patient characteristics and medical interventions in HHOs with iHEC

The median patient age was 50 years (range 17–85), and 44 patients (81%) were male. Traumatic causes (including secondary asphyxia, i.e., avalanche and drowning) were the reason for intubation in 32 patients (59%), whereas 22 intubations (41%) were performed due to acute medical conditions. The median NACA score was 6 (range 5–7) and the median Glasgow coma scale (GCS) score prior to intubation was 3 (range 3–15).

Twenty-four iHEC patients (44%) were intubated before arrival of the RRT by either another HEMS physician or a ship doctor. Drug-assisted rapid sequence intubation (RSI) was performed in 31 patients (57%). Other advanced medical interventions on-scene included thoracostomy (n = 4, 7%), vasoactive drug infusion (n = 3, 6%) and blood product transfusion (n = 2, 4%). CPR was performed on 25 patients (46%), of whom 10 (40%) obtained ROSC before hoisting. Of the 15 patients with persistent cardiac arrest, 13 patients (87%) were hoisted with an ongoing mechanical chest compression device (mCPR). Two patients in cardiac arrest had a period of no-flow during the hoist procedure. Eleven cardiac arrest cases (44%) were presumed to be of primary coronary origin. Although it was registered in a few patients, the occurrence of hypothermic cardiac arrest (or core temperature) was not consistently documented in the written reports. The median on-scene time for the RRT was 23 min (range 1–74).

During hoisting, 34 patients (63%) were ventilated manually with a self-inflating bag, whereas 20 (37%) were connected to an automatic ventilator. While one base only practiced manual ventilations, another base used an automatic ventilator in 83% of the cases. The most frequently used ventilator was a compact battery powered turbine ventilator.

Monitoring of vital parameters during hoisting varied from none (n = 4, 7%), to fully monitored patients with peripheral oxygen saturation (SpO_2_), electrocardiogram (ECG, capnography and IABP (n = 2, 4%). Thirty-nine patients (72%) were documented to having ≥ 1 vital signs (capnography or SpO_2_) monitored during hoisting and 25 (46%) had ≥ 2 vital signs monitored. The database registries were nondescriptive for 10 cases (19%).

Table 3 presents an overview of patient characteristics and interventions in HHOs with iHEC.

**Table 3:** Patient characteristics and medical interventions in HHOs with iHEC 2011—April 2024 (n = 54). Only interventions that could be verified to be delivered on-scene are included

Variable	n	%
Age, median years (range)	50 (17–85)	
< 18 years	1	2
> 80 years	2	4
Gender		
Male	44	81
Female	10	19
Diagnosis		
Trauma	32	59
Medical	22	41
NACA Score (National Advisory Committee for Aeronautics)		
0—4	0	0
5—Injuries/diseases with acute threat to life	7	13
6—Injuries/diseases transported after successful resuscitation of vital signs	44	81
7—Lethal injuries or diseases (with or without resuscitation attempts)	3	6
GCS score before intubation		
GCS 3	32	59
GCS 4–8	14	26
GCS 9–14	6	11
GCS 15	2	4
Procedures and interventions prior to hoisting		
Rapid sequence intubation (RSI)	31	57
Cardiopulmonary Resuscitation (CPR)	25	46
Mechanical chest compression device (mCPR)	13	24
Point of care ultrasound (POCUS)	6	11
Tube thoracostomy	4	7
Vasoactive drug infusion	3	6
Blood product transfusion	2	4
Invasive arterial blood pressure (IABP)	2	4
Central venous catheter	2	4
Ventilation strategy during hoisting		
Manual ventilation	34	63
Automatic ventilation	20	37
Patient monitoring during hoisting		
Continuous monitoring of ≥ 1 vital sign	39	72
Continuous monitoring of ≥ 2 vital signs	25	46
No monitoring	4	7
Unknown/missing	10	19

### Adverse events in HHOs with iHEC

Four adverse events related to the hoist procedures were identified. In two of the cases, stretcher rotation resulted in periods of suboptimal manual ventilation. In the third case, the helicopter downwash resulted in disconnection between the manual ventilation bag and the endotracheal tube. The physician was here unable to reconnect the bag before entering the helicopter cabin, at which point the SpO_2_ had decreased to approximately 80%. In the fourth case, a brief disconnection between the filter and tube occurred during the loading of an iHEC patient into the helicopter cabin. None of these events were deemed by the physician to affect patient outcome.

### Patient outcome in HHOs with iHEC

Twenty-eight patients (52%) survived to hospital discharge. Among the survivors, seven (25%) presented with initial cardiac arrest, of whom six obtained return of spontaneous circulation (ROSC) before hoisting. The last cardiac arrest survivor received continuous mCPR to an extracorporeal life support (ECLS) centre. Twenty-four iHEC patients (44%) did not survive to hospital discharge, and the outcome was unknown for two patients (4%).

## Discussion

Helicopter hoisting of iHEC is a complex operation. During a few crucial minutes, all six crew members are involved simultaneously in the process of safely extricating a critical care patient. This requires mutual understanding and integration of different procedures and clear communications, while at the same time maintaining physiological optimalization and continuous ventilatory support [1, 2].

HHOs with iHEC is an infrequent procedure in our service, comprising only 2% of all hoist operations with HEC and 3% of our prehospital intubations. A median NACA score of 6, a median GCS of 3 and a survivability rate of 52% indicate not only that the patients were critically ill, but also that the threshold for intubation prior to hoisting was high.

The incidence reported in this study is consistent with that reported previously. Despite the low incidence of iHEC patients, our data indicate an increased frequency of HHOs with both HEC and iHEC patients over time. The factors contributing to this development are likely multifactorial. Between 2020 and 2024 the AW101 replaced the Sea King helicopter on the 330 Sqn bases. Due to stricter landing zone requirements in terrain and rural areas, HHOs might therefore be favoured more often compared to the Sea King era. In addition, the physicians have taken an increasingly active role in HHOs. In 2019, the 330 Sqn started a program called SAR Ground Operations (SAGO), with the intention to train the physicians in rescue techniques and risk awareness to better assist the RS in rescue operations. The SAGO program comprises 21 days of basic training (rescue techniques on rock, snow, ice, alpine terrain, urban SAR and swift water) followed by a yearly recertification week combining medical and technical aspects of HSAR operations. While the physicians were hoisted in 23% of HHOs in 2011, they were hoisted in 56% of HHOs in 2023. This development may offer patients accessed by HHOs increased possibilities for onsite advanced life support.

### Ventilation strategies in HHOs with iHEC

There is limited information in the literature on optimal ventilation strategies during hoist extrication of intubated patients. It appears to be most common to utilize manual bag-tube ventilation, which was also most prevalent in our data. For some services, and in certain situations, it may be the only option. A self-inflatable ventilation bag has low weight, it works well in most conditions and provides tactile feedback to airway resistance.

Manual ventilation during HHOs is, however, not without risk. In 2012, Burns et al. described a case where a ventilation bag lost the ability to self-expand when passing through the helicopter downwash [13]. We identified in our data a case of disconnection between the ventilation bag and the endotracheal tube due to downwash, and two cases of suboptimal manual ventilation due to stretcher rotation.

A review article from Pietsch et al. indicated that, given the lack of other data, manual ventilation during hoisting should be the preferred strategy [1]. The authors feared that disconnection or malfunction of an automatic ventilator could be neither perceived nor managed during hoisting. In a response to this article, Hollott argued that automatic ventilation is superior to manual ventilation with a low risk of malfunction and should be the gold standard [16]. His position was mainly based on a previous mannequin study. Hollot and colleagues demonstrated here that, compared to manual ventilation in iHEC, automatic ventilation provided more stable ventilation and airway pressures and hence presumably more stable oxygenation and arterial carbon dioxide levels [17].

In our experience, the use of an automatic ventilator in HHOs with iHEC increases both the quality of ventilation and safety aspects for the patient and the rescuers. When a ventilator is used, the rescuer is free to maintain situational awareness throughout the hoist operation, including monitoring of vital signs and stretcher management. Unexpected events are more easily managed without affecting ventilation. The risk of airway disconnection, tube dislocation or kinking is considerably reduced when the connectors and tubing are protected inside a rescue bag. Loading of the patient into the helicopter can be performed in a timely manner and with increased safety margins. In addition, oxygen consumption is significantly lower than that of manual ventilation, which is particularly true for battery-powered turbine ventilators. This might be critical in complex environments with limited access to oxygen.

### Patient monitoring in HHOs with iHEC

Six patients (32%) in the first half of the study period had continuous monitoring of two or more vital signs compared to 19 patients (54%) during the last half of the study. In the last period, two patients had a level of monitoring normally found in an advanced medical care environment. The reason for this development may be related to better prehospital monitoring equipment in recent years. Although we recommend that iHEC patients be fully monitored with wave-form capnography, SpO_2_, blood pressures and ECG, we recognize that this is not always possible when environmental threats or other risk factors mandate a rapid extrication.

### Practical aspects of medical management in HHOs with iHEC

**Fig. 4:**
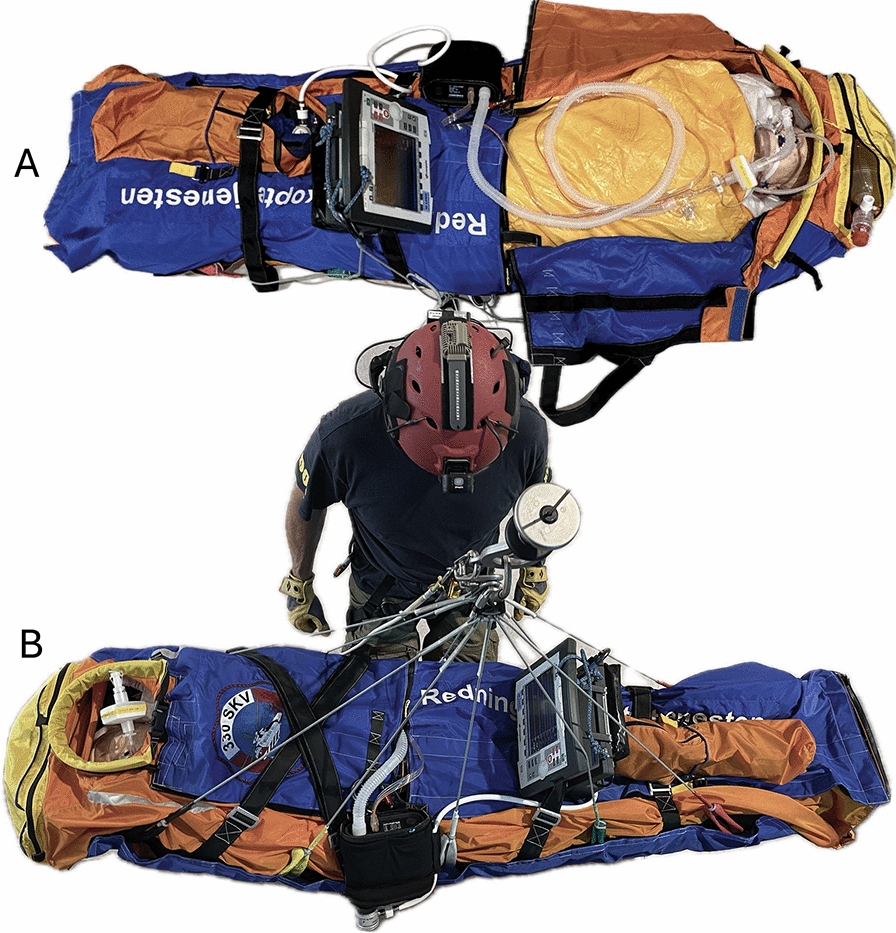
The recommended set-up for hoisting iHEC in the RNoAF 330 Sqn. **A**. The monitor cables are concealed under the hypothermia bag. The respiratory tubes are placed over the patient’s chest and connected to the endotracheal tube via an extender swivel. The oxygen cylinder is in placed in a designated compartment. A self-inflatable ventilation bag is accessible from the cranial pocket of the rescue bag. IV access is available through a 100 cm extension to the patient’s neck. **B**. During the hoist procedure both the monitor and ventilator displays are in clear view of the physician. Turbine ventilators need to be placed with access to fresh air and without the possibility of air inlet occlusion

In our service we recommend that the monitor and ventilator is secured on the rescue bag as shown in Fig. 4. Before hoisting, FiO_2_ should be adjusted to the maximum level until the patient is safely established in the helicopter cabin. A self-inflatable ventilation bag should be readily accessible in the hood or a cranial pocket of the rescue bag in case of ventilator failure. The physician accompanying the patient should have relevant drugs and sedatives prepared on their body, and easy access to an IV-line.

### Mechanical chest compressions in HHOs

**Fig. 5:**
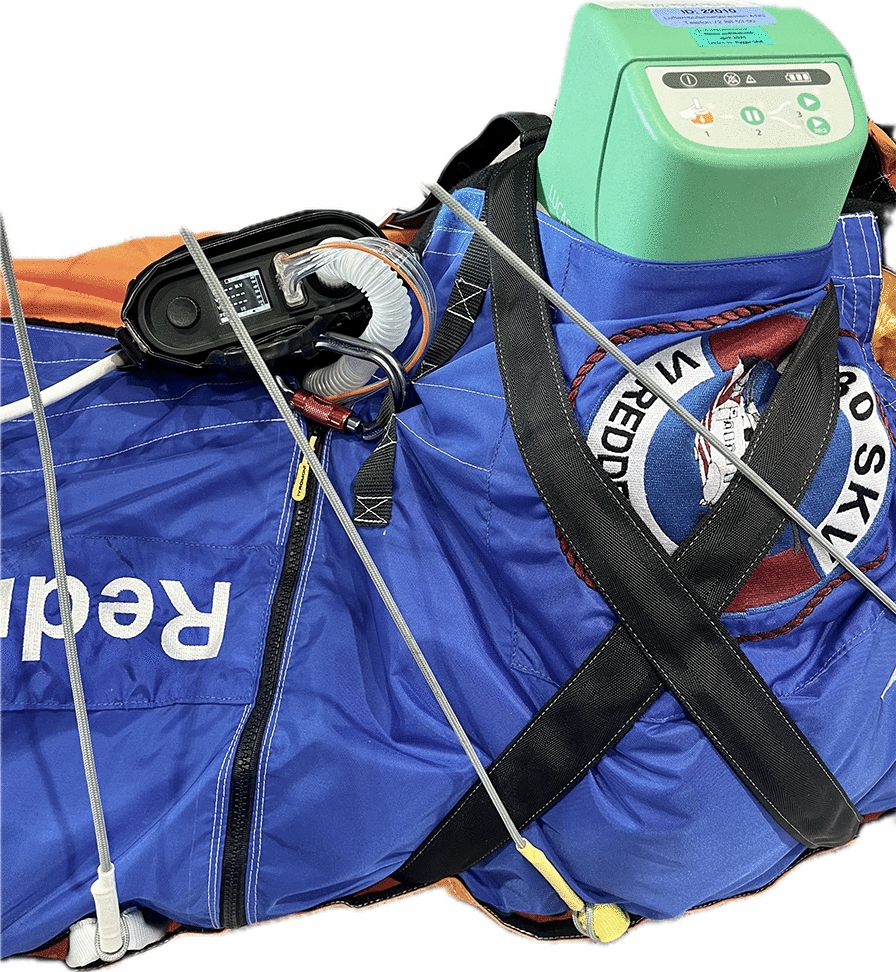
The LUCAS device is secured by Velcro and cross-strapping. For patients with narrow chests, we speculate if placing one or both arms parallel to the body might be beneficial in preventing lateral movement within the LUCAS device during hoisting. The automatic ventilator provides acceptable ventilation until the patients is established in the helicopter cabin and manual ventilation can be resumed

Mechanical chest compression devices are increasingly common in HEMS and HSAR [18]. These devices increase the quality of uninterrupted chest compressions from the accident scene to the hospital, especially over complex terrain and during transport [19–21]. We have only found one reported case of mCPR during a HHO [19]. In our material we identified 13 cases, in which one drowned victim in hypothermic cardiac arrest survived with a good neurological outcome. Of note, the staging of hypothermia was generally poorly described in our data. It could be argued that for patients with core temperature < 28 °C, the rescue team could utilize the principals of intermittent CPR (iCPR) as the no-flow time would statistically be < 5 min during the hoist procedure [22]. While this might be an applicable solution when presented with no other option, this approach leaves little redundancy for unexpected events resulting in a delayed hoist extrication. In our experience, the LUCAS mechanical chest compression device (LUCAS 2, Jolife AB, Lund, Sweden) can be fixated securely in a helicopter rescue bag in order to deliver continuous chest compressions during hoisting (Fig. 5). We recommend that the compressions are paused briefly if the patient position deviates from the horizontal plane, i.e. loading into- and out of the helicopter, and not resumed until correct piston placement is confirmed.

### Strengths

Analysis of helicopter rescue operations involving several operative phases and sequences of medical management is challenging. This study highlights some of these challenges and might provide useful information for future prospective studies.

## Limitations

Baseline parameters were missing or unknown for several missions. This is probably partly due to major events and disaster situations where multiple patients were evacuated in rapid succession to local EMSs. For instance, when the cruise ship Viking Sky lost engine power close to shore during a storm in March 2019, 474 people were evacuated by HHOs. During a rain flood with multiple landslides in 2023, 86 people were rescued. In neither of these situations were individual patient reports recorded.

For HHOs, the electronic search strategy relied on keywords written in the medical reports. The inability of the databases to apply direct harvest of the chosen parameters represents an uncertainty to whether we have identified all HHOs with iHEC. For some missions, the written reports were scarce, requiring supplemental information from the mission physicians when possible. Moreover, for several missions, it was difficult to ascertain in what phase of the rescue the registered medical interventions were performed. Studies on HHOs with iHEC are few in number and small in volume. For future studies and continuous improvement, our database registries should be improved to better distinguish between medical interventions performed in different phases of a helicopter rescue and better document any occurrence of adverse events.

Finally, the RNoAF 330 Sqn utilizes larger helicopters than those normally found in civilian HEMS. Services with smaller aircrafts or different mission profiles may recognize different challenges and solutions to those described in this study.

## Conclusions

Helicopter hoist operations with intubated patients are rare but increasingly frequent events in our service. Prehospital developments in ventilation strategies and patient monitoring possibilities may facilitate improved patient safety in austere locations. As iHEC operations are both infrequent and complex, they should be governed by dedicated operating procedures and practiced regularly. If applied conscientiously, we conclude that HHOs with iHEC represents a favourable option in situations where terrestrial evacuation is associated with either a significant time-delay or additional risk factors to the patients or the rescuers.

## Data Availability

No datasets were generated or analysed during the current study.

## Abbreviations

CPR: Cardiopulmonary resuscitation
ECLS: Extracorporeal life support
HSAR: Helicopter search and rescue
HEMS: Helicopter emergency medical service
HHO: Helicopter hoist operation
HEC: Human external cargo
IABP: Invasive arterial blood pressure
iHEC: Intubated human external cargo
mCPR: Mechanical CPR, i.e. CPR assisted by a mechanical chest compression device
ROSC: Return of spontaneous circulation
RS: Rescue specialist with nurse or paramedic background
RSI: Rapid sequence intubation
RRT: Rescue- and resuscitation team (RS + physician)
